# Impact of habitual physical activity and age on T cell metabolism and activation via β_2_-adrenergic receptor modulation

**DOI:** 10.3389/fragi.2026.1869552

**Published:** 2026-10-20

**Authors:** Amanda V. Sardeli, Ana Ramkrapes, Jakob Boyes-Jensen, Jon Hazeldine, Helena Batatinha, Niharika A. Duggal, Davide Calebiro, James E. Turner

**Affiliations:** 1 Department of Inflammation and Ageing, School of Infection, Inflammation and Immunology, College of Medicine and Health, University of Birmingham, Birmingham, United Kingdom; 2 National Institute for Health and Care Research (NIHR), Biomedical Research Centre, Birmingham, United Kingdom; 3 Laboratory of Exercise Physiology (FISEX), University of Campinas, Campinas, Brazil; 4 Department of Kinesiology, School of Health and Human Sciences, University of North Carolina Greensboro, Greensboro, NC, United States; 5 Department of Metabolism and Systems Science, School of Medical Sciences, College of Medicine and Health, Birmingham, United Kingdom; 6 School of Sport, Exercise and Rehabilitation Sciences, College of Life and Environmental Sciences, University of Birmingham, Birmingham, United Kingdom

**Keywords:** adrenergic, aging, exercise, immunosenescence, lymphocyte, metabolism, physical activity, T cell

## Abstract

**Background:**

Ageing impairs T cell function and responses to vaccines and infections. Effective T cell function requires rapid metabolic switching towards glycolysis, which becomes dysregulated with age. We have shown that physically active older adults have T cells that are more functional and metabolically fit. As exercise acutely elevates adrenaline and mobilises T cells via β_2_-adrenergic receptors (β_2_-AR), we hypothesise that habitual physical activity (PA) preserves β_2_-AR-mediated metabolic stimulation in T cells from older adults.

**Methods:**

Older adults (≥65 years) with habitually high PA (HPA, n = 6) or habitually moderate PA (MPA, n = 6) were compared with young controls (18–35 years, n = 6). T cells were exposed to the β-agonist isoproterenol for 30 min to mimic acute exercise-like adrenergic stimulation, with or without the β_2_-AR blocker ICI-118,551. Following 1 h of rest, T cells were stimulated for 4 h *in vitro* with PMA and ionomycin. T cell activation (CD69), glucose transporter-1 (Glut1), intracellular cAMP concentrations under isoproterenol stimulation, and β_2_-AR mRNA expression were assessed.

**Results:**

Older adults exhibited impaired CD8^+^ T cell activation and β_2_-AR blockade inhibited activation in all groups. Glut1 expression in CD8^+^ T cells was higher in older MPA than young, but did not differ from older HPA. In CD8^+^ T cells that were not stimulated with PMA and ionomycin, β_2_-AR blockade (with β-AR stimulation) increased Glut1 expression in all groups compared with β-AR stimulation alone, and this increase was greater in older HPA than MPA (p = 0.043). No group differences were observed in β_2_-AR mRNA expression or isoproterenol-induced β-AR cAMP responsiveness.

**Conclusion:**

These exploratory findings indicate that individuals with higher levels of habitual PA have lower age-associated impairments in CD8^+^ T cell activation and metabolic switching in response to β stimulation. Interestingly, β_2_-AR blockade did not fully abrogate β stimulation, therefore future studies should investigate a potential influence of β_1_-AR in this modulation, to generate definitive conclusions.

## 1 Introduction

Optimal T cell metabolism and function are essential for older adults to sustain a robust immune response. For example, impairments in T cell proliferation are associated with weakened vaccine responses (Herrero-Fernández et al., 2019), increased infection severity (Autaa et al., 2025), and reduced pathogen control (Pahar et al., 2012). Effective T cell functions depend on the activity of specific metabolic pathways, for example, switching towards glycolytic activity upon antigen stimulation and reducing glycolytic activity and increasing oxidative phosphorylation (OXPHOS) when infections are resolved (Voss et al., 2021). This metabolic flexibility is altered with aging, where the activity of different metabolic pathways seems to be more constant across different cellular states (Møller et al., 2022; Quinn et al., 2023). For example, higher levels of habitual physical activity (PA) in older adults are associated with improvements in T cell metabolic profiles, under both unstimulated and stimulated conditions (Hazeldine et al., 2025). During activation, T cells from highly physically active older adults displayed an enhanced ability to upregulate glycolysis, a metabolic shift that is associated with dampened hyperinflammatory responses and greater proliferation capacity (Hazeldine et al., 2025).

The mechanisms through which habitual PA shapes T cell metabolism remain unclear, but adrenergic signalling is a plausible mediator of this metabolic regulation, given its established role in regulating cellular energetics across tissues pathways, increasing energy availability during stress responses (Ciccarelli et al., 2013; Perez, 2021). During exercise, adrenaline binds to adrenergic receptors on a variety of cells to regulate immunological and metabolic processes. Adrenaline-induced changes to metabolism are well known in muscle and adipose tissue, where mitochondrial activity is increased, triggering changes in mitochondrial morphology and function, potentially by the cAMP-PKA pathway (Evans et al., 2015; Voltarelli et al., 2021). However, the role of adrenergic receptors on immune cells is being increasingly well-defined. Adrenergic receptors are divided between α- and β-subtypes (α1A, α1B, α1D, α2A, α2B, α2C, β_1_, β_2_, and β_3_). Immune cells predominantly express β2-adrenergic receptors (Williams et al., 1976; Simpson et al., 2021), which are G protein-coupled transmembrane receptors (GPCRs) that undergo conformational changes upon agonist binding, thereby initiating downstream intracellular signalling pathways. Activation of the β2-adrenergic receptor stimulates adenylyl cyclase (AC), leading to increased cyclic adenosine monophosphate (cAMP) production and subsequent activation of protein kinase A (PKA). Adrenergic signalling modules several intracellular pathways which results in the activation of various transcription factors (Kohm and Sanders, 2000). The expression levels of β_2_-adrenergic receptors is not static; varying with immune cell subtype, genetic background, activation state, and the surrounding cytokine environment (Bruck et al., 2003). For instance, CD8^+^ T cells exhibit a greater surface density of β_2_-adrenergic receptors compared with CD4^+^ T cells (Landmann, 1992). Overall, it is well known that during exercise, T cell mobilisation is regulated by β_2_-adrenergic receptor binding (Simpson et al., 2021) and it is becoming increasingly recognized that β_2_-adrenergic signaling also regulates T cell metabolism. Indeed, studies in circulating immune cells, have shown that systemic β-adrenergic blockade during exercise blunts exercise training-induced changes in T cell proliferation and cytokine production in old mice (Kohut et al., 2004). Therefore, we speculate that adrenaline-induced stimulation of mitochondrial activity with repeated exercise bouts during exercise training might modify cellular machinery (i.e., facilitate substrate access) to support energy production by T cells in the longer-term.

This hypothesis-generating human study explored the modulatory effects of β_2_-AR stimulation on T cell activation and metabolic regulation in the context of aging and habitual PA. Our hypothesis was that higher levels of habitual PA would be associated with enhanced β_2_-AR response in T cells among older adults, improving cell activation and efficient substrate utilization.

## 2 Materials and methods

### 2.1 Study participants

Ethical approval for this study was granted by the HRA and Health and Care Research Wales HRCW Approval (IRAS 301974) and written consent was obtained from individuals prior to participation. Participants were categorized into two age groups: a young group (18–35 years) and an older group (65 years and above). The older group of participants was split into high habitual PA levels (HPA) and moderate habitual PA levels (MPA), according to their Moderate-to-vigorous activity (MVPA), assessed by 7-days of accelerometery, as shown in Supplementary Figure S1 (Hazeldine et al., 2025). All participants were in good health defined as being free from infection, disease, and immunosuppressive medication. Younger participants did not have physical activity assessed by accelerometery, and the comparison between their PA levels with the older adults was made via a general health questionnaire, where they reported the typical number of minutes engaged with “light”, “moderate” and “vigorous” PA each week during the last year, as well as the typical number of hours spent being sedentary each week during the last year.

### 2.2 Study design

We used a series of *in vitro* experiments to test the influence of β-adrenergic stimulation with isoproterenol with or without a β_2_-AR blockade (ICI-118,551) on markers of T cell activation and metabolism, including a glucose transporter (Glut1), glucose utilization and lipid accumulation at rest and upon activation (with PMA and Ionomycin). In these experiments, a 30-min adrenergic stimulation was used to mimic the short increase in adrenaline with exercise (Voltarelli et al., 2021), and avoiding the harmful effects of longer stimulations that would be more representative of chronic stress (Simpson et al., 2021). Following 1 h of rest, cells were stimulated with PMA and ionomycin, to represent a challenge that could happen in the post-exercise period. In addition, we estimated T cell β_2-_AR receptor expression via the transcriptional abundance of the ADRB2 gene, and isoproterenol-induced β-AR cAMP responsiveness, via intracellular cAMP concentrations, in the different age and PA groups. It is noteworthy that qRT-PCR provides an indirect indication of receptor expression rather than a direct measurement of receptor protein abundance or cell-surface receptor density.

### 2.3 PBMC isolation and cryopreservation

Peripheral blood (40 mL) was collected into heparinised vacutainers (Scientific Laboratory Supplies). Next, samples were diluted 1:1 with RPMI- 1640 medium supplemented with L-glutamine, penicillin, and streptomycin (Life Technologies Limited). The diluted blood was layered over 6 mL Ficoll-PaqueTM PLUS (GE Healthcare) and centrifuged at 400 *g* for 30 min at room temperature (RT) without brake or acceleration. The PBMC layer was collected and transferred into universal tubes containing MACS® buffer (Miltenyi Biotec, Surrey, UK) and centrifuged at 300 *g* for 10 min at RT with full brake. The resulting pellet was resuspended in MACS buffer and washed again by centrifugation under the same conditions. For cryopreservation, cells were resuspended in freezing medium consisting of 10% DMSO (Sigma-Aldrich) in heat-inactivated fetal calf serum (ThermoFisher). A total of 1 mL of freezing medium was used to resuspend the final pellet, and 500 μL aliquots were transferred into 1 mL cryovials (Greiner Bio-One). Cryovials were placed into a Mr. Frosty freezing container (ThermoFisher) and stored at −80 °C for controlled-rate freezing. Cryopreservation was employed to allow batch processing of samples, ensuring consistency across downstream analyses.

### 2.4 PBMC thawing and T cell isolation

PBMCs were thawed at 37 °C and added to a universal tube containing 10 mL of warm phosphate buffered saline (PBS) solution prepared from tablets (P4417, Sigma-Aldrich). This PBMC PBS solution mix was centrifuged at 300 *g* for 10 min at RT and for the experiments that required isolation (β_2_-AR mRNA abundance and cAMP assay), they were resuspended in 1 mL of warm PBS. CD4^+^ and CD8^+^ T cells were negatively selected using the EasySep™ human CD4^+^ and CD8^+^ isolation kit (STEMCELL Technologies) according to the manufacturer’s instructions. Negative selection was chosen to preserve the native activation state and functionality of CD4^+^ and CD8^+^ T cells, which is critical for downstream stimulation and metabolic assays.

### 2.5 T cell purity via flow cytometry

Fifty thousand CD4^+^ and CD8^+^ T cells (diluted in RPMI at 1 × 10^6^/mL) were stained with 1 μL of anti-CD3 (PE-Cyanine7, 0.2 μg/mL, Life Technologies Corp, Clone:UCHT1) and anti-CD4 (Brilliant Violet 421™, 1 μg/mL, BioLegend®, Clone: RPA-T4) and anti-CD8 (PE, 0.24 μg/mL, BioLegend®, Clone: QA18A37) on ice in the dark for 20 min. After incubation, cells were washed with PBS and resuspended in 200 μL PBS for analysis on a BD FACSVIA flow cytometer. Compensation was performed on the same human T cells using single stained controls and applied using BD FACSuite software. Gating was applied on singlets and live lymphocytes, confirming ≥95% purity for both CD4^+^ and CD8^+^ T cells populations.

### 2.6 PBMCs stimulation *in vitro*

PBMCs were thawed at 37 °C and washed in 10 mL of pre-warmed RPMI-1640 medium. Following centrifugation at 300 *g* for 10 min at RT, cells were adjusted to 2 × 10^6^ cells/mL. Aliquots of 2 × 10^5^ cells (100 μL) were distributed into six tubes (Tubes 1–6), each corresponding to a specific treatment condition: tube 1: untreated control, tube 2: isoproterenol (ISO) + PMA/ionomycin + ICI-118,551 (a β_2_-blocker), tube 3: ISO + ICI-118,551, tube 4: ISO + PMA/ionomycin, tube 5: ISO alone, tube 6: PMA/ionomycin. The β_2_-blocker ICI-118,551 was added at a final concentration of 300 nM, and incubated at 37 °C, 5% CO_2_ for 30 min. ISO was then added to achieve a final concentration of 1 μM and incubated for an additional 30 min. Cells were washed with 300 μL PBS, centrifuged at 250 *g* for 5 min at 4 °C, and resuspended in 200 μL RPMI for a 1-h resting period. Next, PMA (final concentration: 50 ng/mL) and ionomycin (final concentration: 500 ng/mL), were added to tubes 2, four and six to stimulate T cell activation. Tubes were incubated for 4 h at 37 °C, before staining with CD69 and Glut1. Thus, the 30 min of ISO exposure was designed to replicate 30 min of exercise followed by 1-h of rest, prior to stimulation with PMA/ionomycin (or control) as detailed in the experimental design (Supplementary Figure S2). ISO was diluted in Hanks’ Balanced Salt Solution (HBSS, Sigma-Aldrich, UK), ICI-118,551, PMA and Ionomycin were diluted in dimethyl sulfoxide (DMSO, Sigma-Aldrich, UK).

Supplementary Figures S3, S4 show an example of CD69 and Glut1 MFI under unstimulated and stimulated conditions for CD4 and CD8^+^. To isolate the effect of β_2_-AR modulation in the results, ΔCD69 and ΔGlut1 median fluorescence intensity (MFI) was calculated as the difference between each treatment and its matched control. For non-stimulated cells, ΔMFI was obtained by calculating tube 5 (ISO alone) minus tube 1 (untreated control) MFI values and tube 3 (ISO + ICI-118,551) minus tube 1 (untreated control) MFI values, to isolate β-adrenergic stimulation and β-adrenergic stimulation with β2 blockade, respectively. For cells stimulated with PMA/ionomycin, ΔMFI was obtained by calculating tube 4 (ISO + PMA/ionomycin) minus tube 6 (PMA/ionomycin) MFI values and tube 2 (ISO + PMA/ionomycin + ICI-118,551) minus tube 6 (PMA/ionomycin) MFI values, to isolate β-adrenergic stimulation upon activation and β-adrenergic stimulation with β2 blockade upon activation, respectively. This approach normalises each donor’s data to their own baseline, thereby highlighting the specific contribution of β_2_-AR stimulation or blockade. PMA and ionomycin were used to induce robust T cell activation by directly stimulating protein kinase C and intracellular calcium flux, bypassing the T cell receptor. This method was selected over anti-CD3/CD28 stimulation due to its faster activation kinetics, which were critical for distinguishing the short-term effects of ISO (that might more closely reflect the acute effects of exercise) from downstream chronic responses. The shorter stimulation window minimizes potential desensitization or adaptation that could mask β_2_-adrenergic signaling outcomes.

### 2.7 T cell activation and metabolism via flow cytometry

T cells from stimulated PBMCs were analysed via flow cytometry. Tubes 1–6 were stained with anti-CD3 (PE-Cyanine7, 0.2 μg/mL, Life Technologies Corp, Clone:UCHT1), anti-CD4 (Brilliant Violet 421™, 1 μg/mL, BioLegend®, Clone: RPA-T4), anti-CD8(PE, 0.24 μg/mL, BioLegend®), anti-CD69 (APC, 4 μg/mL, BioLegend®, Clone: FN50), anti-Glut1 (FITC, 0.75 μg/mL, R&D Systems). Control tubes for metabolic profiling were stained with anti-CD3, anti-CD4, anti-CD8, and anti-CD69 without Glut1. Staining was performed on ice, in the dark for 20 min, followed by a final wash and resuspension in 200 μL PBS for acquisition. Flow cytometry was conducted using a MACSQuant® Analyzer 8 Flow Cytometer (Miltenyi Biotec). The marker CD69 was selected as a rapid, early T cell activation marker, while Glut1 expression reflects activation-induced metabolic reprogramming via increased glucose uptake (Macintyre et al., 2014). Cell-surface Glut1 expression was assessed using a fluorescein-conjugated mouse monoclonal anti-human Glut1 antibody (clone 202915; R&D Systems, FAB1418F), which recognizes an extracellular epitope of human Glut1. These markers were chosen due to their sensitivity to acute stimulation and relevance to T cell activation and metabolism. In addition, we assessed glucose utilization and lipid accumulation in T cells using 2-NBDG (Cat# 11569116, Fisher Scientific Ltd.) and BODIPY™ 493/503 (Cat# 11540326, Fisher Scientific Ltd.), respectively. For 2-NBDG assays, 300 μL of the cell suspension was centrifuged at 1,500 × g for 2 min at RT, resuspended in 200 μL glucose-free RPMI, and stained with 100 μM 2-NBDG, for 30 min. For lipid droplet assessment, 2.6 μL BODIPY™ 493/503 was added to relevant tubes after 3 h of stimulation and RPMI- 1640 medium supplemented with L-glutamine, penicillin, and streptomycin (Life Technologies Limited) was used as vehicle. For surface marker staining, cells were washed and incubated with antibody cocktails prepared in PBS. Compensation controls and fluorescence-minus-one (FMO) controls were to ensure gating accuracy. Flow cytometry data were processed using FlowJo version 10.8.1. Compensation was applied with single-stained controls, and populations were gated sequentially by forward/side scatter, followed by CD3 with either CD4 or CD8 expression. T cells activation (CD69 expression), levels of the Glucose Transporter 1 (Glut1 expression), glucose utilization and lipid accumulation (2-NBDG/BODIPY™ 493/503) were quantified as MFI rather than the percentage of positive cells because their fluorescence were analysed as continuous measures of marker expression or intracellular substrate/lipid content, rather than as binary positive/negative phenotypes (Tangri et al., 2013). MFI is less influenced by outlying events than the arithmetic mean, making it appropriate for detecting shifts in the magnitude of cellular expression or uptake. The frequencies of positive CD69 and Glut1 cells are reported in Supplementary Table S1 and gating strategies are presented in Supplementary Figures S5–S7. Although the cells were not live/dead stained the lymphocytes population were first selected after the forward and side scatter gating immediately after the experiment without the need for fixation or permeabilization.

### 2.8 β_2_-AR mRNA abundance via qRT-PCR

Total RNA was extracted from isolated CD4^+^ and CD8^+^ T cells using the RNeasy Mini Kit (QIAGEN, cat. No. 74104) according to the manufacturer’s instructions. RNA concentrations were quantified using a NanoDrop spectrophotometer. Only samples with an A260/A280 ratio of between 1.8 and 2.0 were used for downstream applications. Quantitative one-step reverse transcription PCR (qRT-PCR) was conducted using the iTaqTM Universal SYBR® Green One-Step Kit (Bio-Rad, cat. No. 1725150). Each 5 μL reaction contained 2.5 μL 2× SYBR Green Reaction Mix, 0.0625 μL iScript Reverse Transcriptase, 300 nM of each primer, 5 ng total RNA, and nuclease-free water. The following primers were used:

β_2_-adrenergic receptor (ADRB2) forward 5′-ACG​CAG​CAA​AGG​GAC​GAG-3′ and reverse 5′- CAC​ACC​ATC​AGA​ATG​ATC​AC-3'; and 18S (reference gene) forward 5′- GTA​ACC​CGT​TGA​ACC​CCA​TT-3′ and reverse 5′-CCA​TCC​AAT​CGG​TAG​TAG​CG-3'. Amplification was performed on the CFX Opus 384 Real-Time PCR System (Bio-Rad) with the following thermal cycling conditions: reverse transcription at 50 °C for 10 min, initial denaturation at 95 °C for 1 min, followed by 40 cycles of 95 °C for 10 s and 60 °C for 30 s. Melt curve analysis was conducted post-amplification to verify the specificity of each reaction. Relative gene expression was calculated using the ΔCt method, with 18S rRNA serving as the internal control. 18S rRNA was chosen as a reference gene due to its stable and high expression in T cells, providing reliable normalisation of β_2_-AR transcript levels across experimental conditions. A no-template control, comprising of gene specific primers and master mix only, was run in triplicate on the PCR plate to ensure no contamination of PCR reagents. The cycle threshold (Ct) value of the target gene for each sample was normalised to that of the reference gene to obtain the ΔCt value.

### 2.9 cAMP concentration via luminescence

Isolated CD4^+^ or CD8^+^ T cells were stimulated with either 10 μM ISO (Sigma-Aldrich, UK) or vehicle control for 2 min at RT, within 100ul total volume. Post-stimulation, cells were lysed using the cAMP-GloTM Lysis Buffer and snap frozen in liquid nitrogen, prior to storage at −80 °C. For analysis, samples were thawed at 37 °C and the assay was performed according to manufacturer’s instructions (cAMP-GloTM Assay, Promega, USA; Cat. No. V1501) using 40 μLof cell lysates. Luminescence was measured using a VarioskanTM LUX Multimode. Concentrations of cAMP across all conditions were normalised to protein content per well using the bicinchoninic acid (BCA) assay (PierceTM BCA Protein Assay Kit, Thermo Scientific, Rockford, IL, USA) according to the manufacturer’s instructions. Protein concentrations of unknown samples were calculated by interpolation from the standard curve with linear regression fit in GraphPad Prism (version 5.03). Supplementary Figure S8 confirms the increase in cAMP. Isoproterenol was applied at 10 μM for cAMP measurements to ensure near-maximal β2-adrenergic receptor occupancy and to generate intracellular cAMP concentrations above the assay detection limit.

### 2.10 Statistical analysis

Statistical analysis was performed using SPSS (version 30.0.0.0; IBM) and results are presented as mean ± standard deviation (SD) in the tables and as median and interquartile range, accompanied by individual data in the graphs. For quantitative PCR data Δ changes (absolute change, by subtracting the initial value from the final value) were calculated relative to untreated control samples. Data was first tested for normality. For comparison between the three groups for single conditions Kruskal-Wallis test, followed by Mann-Whitney U test were used. Mixed-model analysis was performed when analyzing the three groups in 2 different conditions (e.g., inactivated and activated), where subjects were included as random factors and AR(1) model was used and in case of significant group by condition interaction was identified (p < 0.05), Bonferroni *post hoc* was applied. Group effects and condition effects were also reported in the when the was no group by time interaction, as it is a common output from mixed model analysis. Group effects mean that by combining all conditions, the groups are significantly different; whilst condition effects means that by combining all groups, the conditions are significantly different. Statistical significance was defined as p < 0.05. In addition, given the low power of the present study, we consider p-values between 0.05 and 0.1 to be a trend (Curran-Everett and Benos, 2004).

## 3 Results

The characteristics of the participants are presented in Table 1. Small variations in sample size for each analysis occurred due to a limited number of PBMC aliquots per participant and is reported in tables and figure legends. Young participants exhibited similar levels of moderate and vigorous PA as HPA and higher levels of moderate and vigorous PA compared to MPA (assessed by questionnaire). Accelerometery data (only assessed in the older groups) showed that HPA exhibit twice the volume of MVPA than MPA, although both groups met PA recommendations (Bull et al., 2020).

**TABLE 1:** Characteristics of the participants.

Variables	Young (n = 6)	MPA (n = 6)	HPA (n = 6)	Group interaction	Young vs. HPA	Young vs. MPA	HPA vs. MPA
Age	24.3 ± 3.8	75.5 ± 1.4	77.3 ± 4.2	0.003	0.004	0.023	1.000
Sex (number of females)	2	5	4	1*	-	-	-
Body mass (kg)	75.3 ± 20.7	74.8 ± 25.3	64.8 ± 8.6	0.777			
BMI (kg/m^2^)	22.5 ± 4.2	28 ± 5.5	23 ± 2.6	0.124			
MVPA (accelerometry: min/day)	-	71.5 ± 29.1	149.5 ± 22.9	0.004			
Grip strength (kg)	33 ± 7.2	22.7 ± 7.6	26 ± 7.2	0.085	-	-	-
Gait speed (m/s)	1.3 ± 0.2	1.1 ± 0.3	1.3 ± 0.3	0.359	-	-	-
30 Second chair stand	19 ± 6.1	12 ± 5	12.7 ± 3.4	0.078	-	-	-
Physical activity (questionnaire)							
Light PA (h/week)	5.5 ± 1.6	5.8 ± 4.9	8.9 ± 2.9	0.191			
Moderate PA (h/week)	2.1 ± 1.6	0 ± 0	1.7 ± 1.3	0.031	1.000	0.039	0.085
Vigorous PA (h/week)	3.5 ± 3	0 ± 0	1.6 ± 2	0.052	0.645	0.046	0.567
Sedentary time (h/day)	1.2 ± 1.5	1.1 ± 1.4	0.9 ± 0.7	0.887			

Data are shown as mean ± SD. PA, Physical activity; HPA, highly physically active; MPA, moderately physically active; vs., versus; h, hours; n = sample size. Kruskal-Wallis test (Group interaction), followed by Mann-Whitney U test were used. * For sex comparison, Fisher’s exact test was used.

### 3.1 Effects of age and habitual PA on β_2_-AR receptor-mediated T cell activation

Figure 1 shows the activation of T cells between the three groups (young, highly physically active [HPA] and moderately physically active [MPA]) with stimulation and inhibition of T cell β_2_-AR receptors. There was a condition effect (p < 0.001) for CD4^+^ T cell activation (CD69 MFI) with PMA and ionomycin stimulation, but there was no significant group by condition interaction (Figure 1A). For CD8^+^ T cells, there was a significant (p < 0.001) increase in activation (CD69 MFI) with PMA and ionomycin stimulation in the young group only leading to significantly higher expression of CD69 compared to HPA and MPA (p < 0.001 and p = 0.003, respectively, Figure 1B). In the absence of PMA and ionomycin stimulation, β_2_-AR stimulation did not influence expression of (CD69, Figures 1C,D). In PMA and ionomycin stimulated T cells, while β_2_-AR stimulation did not influence the expression of CD69 on CD4^+^ T cells (Figure 1E), there was a condition effect for CD8^+^ T cells (p = 0.011), characterized by a reduction in activation (CD69 MFI) from β-AR stimulation to β-AR stimulation with β_2_-AR blockade (Figure 1F).

**FIGURE 1:**
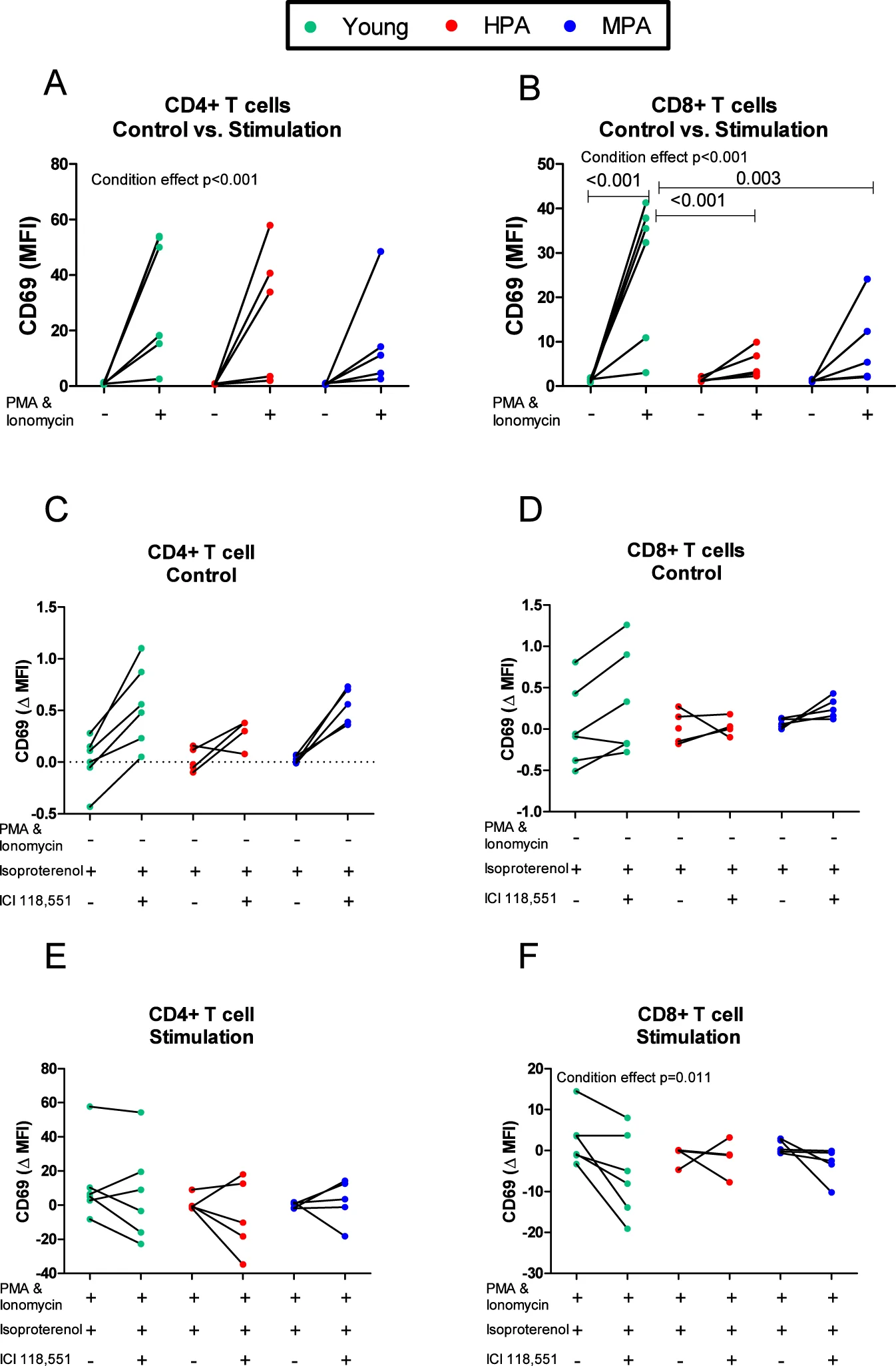
CD69 Expression on T cells (from PBMCs) under the six treatment conditions. **(A, B)**: CD69 expression MFI on CD4^+^ and CD8^+^ T cells with and without PMA/ionomycin stimulation. **(C, D)**: ΔCD69 MFI on CD4^+^ and CD8^+^ T cells calculated as the difference between CD69 expression in control tubes (no PMA/ionomycin stimulation): isoproterenol–untreated control and ICI-118,551 + isoproterenol–untreated control. **(E, F)**: ΔCD69 MFI on CD4^+^ and CD8^+^ T cells calculated as the difference between CD69 expression in stimulated tubes (PMA/ionomycin stimulation): (isoproterenol + PMA/ionomycin) – PMA/ionomycin alone and (ICI-118,551 + isoproterenol + PMA/ionomycin) – PMA/ionomycin alone. Data are shown as median fluorescence intensity (MFI) for each donor (Y: n = 6; HPA: n = 5, MPA: n = 5). Y = Young, HPA = high physical activity, MPA = moderate physical activity. Mixed-model analysis was performed in 2 different conditions (e.g., inactivated and activated), where subjects were included as random factors and AR(1) model was used and in case of significant group by condition interaction was identified (p < 0.05), Bonferroni *post hoc* was applied. Group effects and condition effects were also reported in the when the was no group by time interaction, as it is a common output from mixed model analysis.

### 3.2 Effects of age and habitual PA on β_2_-AR receptor-mediated T cell metabolic response

Given that T cell activation is tightly coupled to metabolic reprogramming, the expression of the glucose transporter Glut1 was assessed as a readout of activation-induced metabolic reprogramming via increased glucose uptake (Macintyre et al., 2014). Upon PMA and ionomycin stimulation, T cells shift towards increased glycolytic metabolism to support their energy and biosynthetic demands. Therefore, examining Glut1 surface expression provided an assessment of whether β_2_-AR modulates the metabolic phenotype of CD4^+^ and CD8^+^ T cells under the same treatment conditions. Condition effects showed an increase in Glut1 expression with PMA and ionomycin stimulation in CD4^+^ (p = 0.002, Figure 2A) and in CD8^+^ T cells (p < 0.001, Figure 2B). There was a group effect in CD8^+^ T cells (Figure 2B), in which Glut1 expression was higher in MPA than young (p = 0.018) and tended to be higher in HPA than young (p = 0.063). In the absence of PMA and Ionomycin stimulation, β-AR stimulation did not regulate Glut1 expression in CD4^+^ T cells (Figure 2C). All groups exhibited increased Glut1 expression in CD8^+^ T cells with β_2_-AR blockade (all p < 0.001), with a significantly higher increase observed in HPA than MPA (p = 0.043), and a trend to a higher increase in HPA than young (p = 0.073), although not significant. Under PMA and ionomycin stimulation in CD4^+^ T cells, there was a trend for a condition effect (p = 0.063, not significant), showing a reduction in delta Glut1 from the β stimulated condition to β_2_-AR blockade (Figure 2E). The group effect showed a trend for delta Glut1 in HPA to be lower than MPA (p = 0.088, not significant). Under PMA and ionomycin stimulation, CD8^+^ T cells underwent a larger increase in Glut1 with β_2_-AR blockade compared to β stimulation only (Figure 2F). Supplementary Figure S9 shows Glut1 expression under the same conditions only within CD69^+^ cells. There was a significant group effect (p = 0.043) in which activated (CD69^+^) CD4^+^ T cells under PMA and ionomycin stimulation among the MPA group tended to have higher Glut1 expression than HPA overall (p = 0.058) (Supplementary Figure S9E).

**FIGURE 2:**
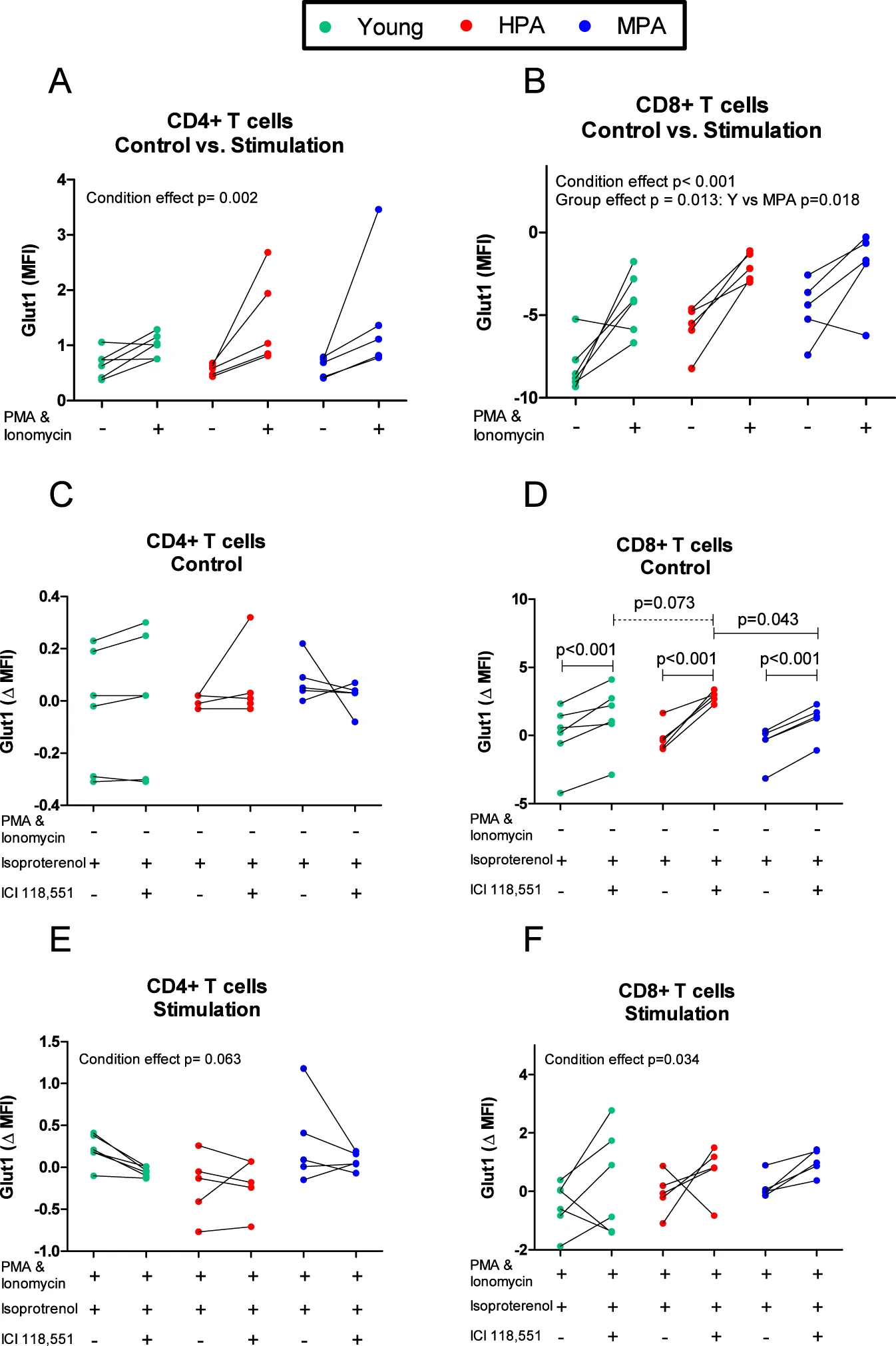
Glut1 expression on T cells (from PBMCs) under the six treatment conditions. **(A, B)**: Δ Glut1 expression MFI on CD4^+^ and CD8^+^ T cells with and without PMA/ionomycin stimulation. **(C, D)**: Δ Glut1 MFI on CD4^+^ and CD8^+^ T cells calculated as the difference between Glut1 expression in control tubes (no PMA/ionomycin stimulation): isoproterenol–untreated control and ICI-118,551 + isoproterenol–untreated control. **(E, F)**: Δ Glut1 MFI on CD4^+^ and CD8^+^ T cells calculated as the difference between Glut1 expression in stimulated tubes (PMA/ionomycin stimulation): (isoproterenol + PMA/ionomycin) – PMA/ionomycin alone and (ICI-118,551 + isoproterenol + PMA/ionomycin) – PMA/ionomycin alone. Glut1 surface expression was then assessed by flow cytometry. Data are shown as median fluorescence intensity (MFI) for each donor (Y: n = 6; HPA: n = 5, MPA: n = 5); vs.: versus. Y = Young, HPA = high physical activity, MPA = moderate physical activity. Mixed-model analysis was performed in 2 different conditions (e.g., inactivated and activated), where subjects were included as random factors and AR(1) model was used and in case of significant group by condition interaction was identified (p < 0.05), Bonferroni *post hoc* was applied. Group effects and condition effects were also reported in the when the was no group by time interaction, as it is a common output from mixed model analysis.

### 3.3 Effects of age and habitual PA on T cell glucose utilization and lipid accumulation

Building on the findings that β_2_-AR signaling influenced Glut1 expression and thus glucose transporter availability, the next step was to directly assess functional (during activation) glucose utilization (via 2-NBDG) and lipid accumulation (via BODIPY™) in T cells in PMA and ionomycin stimulated and unstimulated T cells (Figures 3A–D). There was a trend for an increase in glucose utilization in CD4^+^ T cells from the MPA group with PMA and Ionomycin stimulation, but this was not statistically significant (p = 0.088, Figure 3A), requiring further validation, while there was no effect in CD8^+^ T cells (Figure 3B). Lipid accumulation (BODIPY™) was not altered with PMA and ionomycin stimulation in CD4^+^ T cells (Figure 3C), but a condition effect (p = 0.010) showed that lipid accumulation (BODIPY™) was reduced with PMA and ionomycin stimulation (Figure 3D).

**FIGURE 3:**
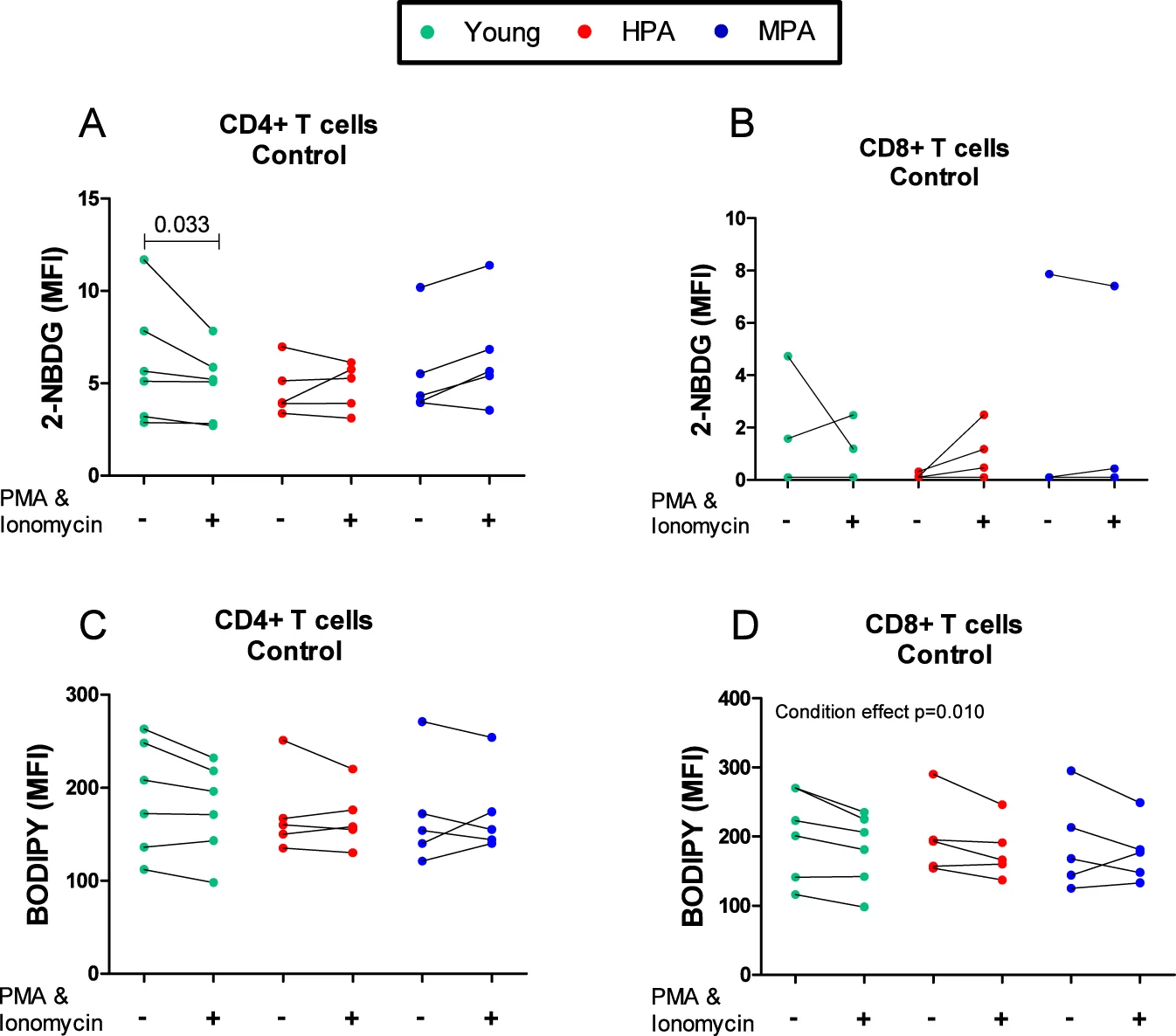
Glucose utilization and lipid accumulation in resting and activated T cells (from PBMCs) across donor groups. **(A)**: 2-NBDG in CD4+ T cells; **(B)**: 2-NBDG MFI in CD8+ T cells; **(C)**: BODIPY MFI in CD4+ T cells; **(D)**: BODIPY MFI in CD8+ T cells. Peripheral blood T cells were stimulated with PMA/ionomycin for 4 h, with either 2-NBDG (100 μM, final hour) added in glucose-free RPMI to assess glucose utilization, or BODIPY™ added to assess neutral lipid accumulation. CD4^+^ and CD8^+^ T cells were identified by surface staining prior to flow cytometric analysis. Data are shown as median fluorescence intensity (MFI) for each donor (Y: n = 6; HPA: n = 5, MPA: n = 5). Y = Young, HPA = high physical activity, MPA = moderate physical activity. Mixed-model analysis was performed in two different conditions (e.g., inactivated and activated), where subjects were included as random factors and AR(1) model was used and in case of significant group by condition interaction was identified (p < 0.05), Bonferroni *post hoc* was applied. Group effects and condition effects were also reported in the when the was no group by time interaction, as it is a common output from mixed model analysis.

### 3.4 Effects of age and habitual PA on the mRNA abundance of T cell β_2_-AR receptors

We next examined whether β_2_-AR mRNA was different with age and PA. There were no significant differences between CD4^+^ and CD8^+^ T cells, nor between donor groups stratified by age (young vs. older) or PA level HPA vs. MPA (Figures 4A,B).

**FIGURE 4:**
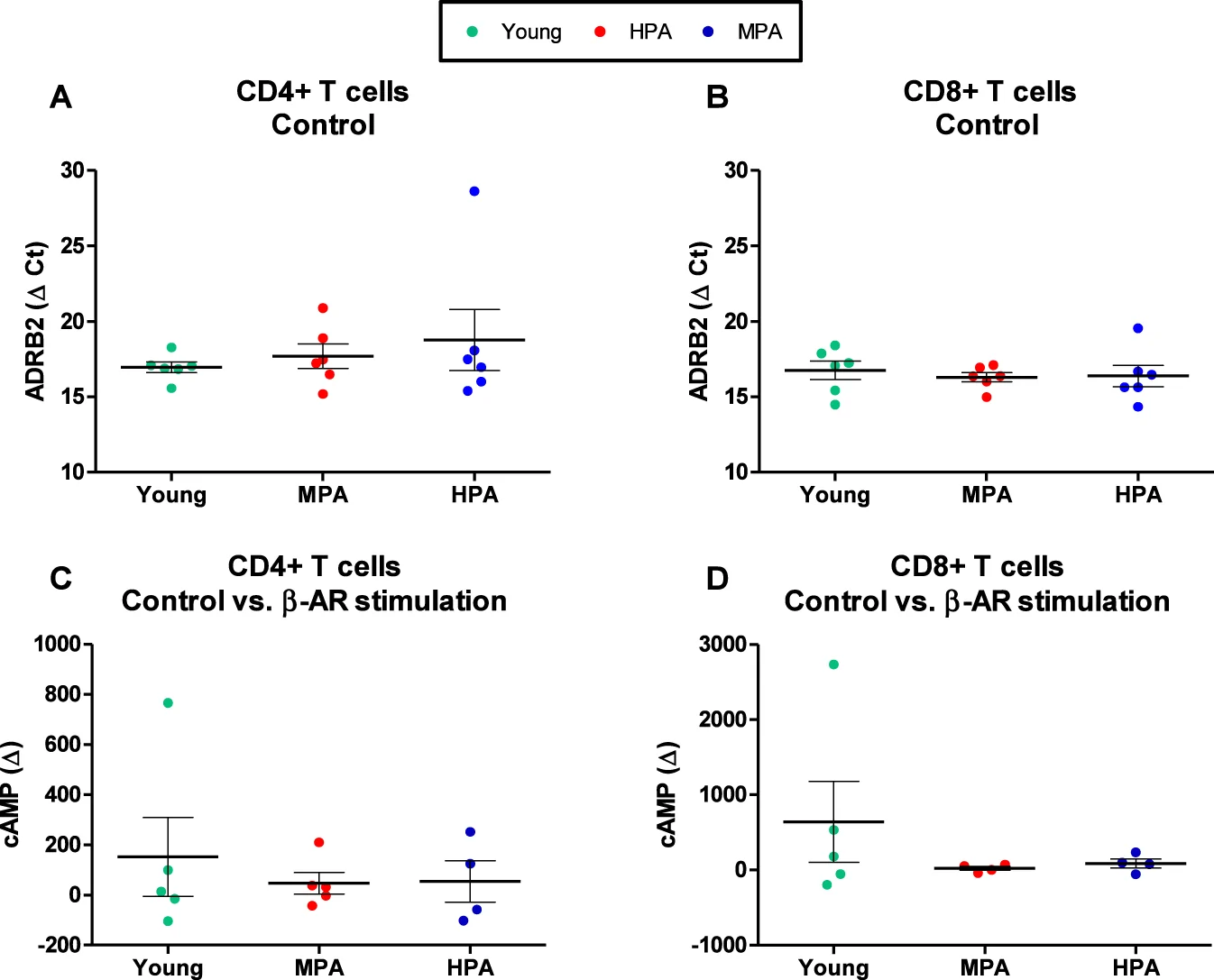
*β*_2_-AR mRNA expression and isoproterenol-induced β-AR cAMP responsiveness from isolated CD4^+^ and CD8^+^ T cells across donor groups. **(A)**: ADRB2 ΔCt in CD4+ T cells under control condition; **(B)**: ADRB2 ΔCt in CD8+ T cells under control condition; **(C)**: cAMP Δ between control and β-AR stimulation in CD4+ T cells; **(D)**: cAMP Δ between control and β-AR stimulation in CD8+ T cells. ADRB2 Δ Ct (the difference between the cycle threshold (Ct) of ADRB2 gene and the Ct of 18S) via Quantitative one-step reverse transcription PCR (qRT-PCR) in isolated CD4^+^ and CD8^+^ T cells from young and older adults in the MPA and HPA groups. *T cells* isoproterenol-induced β-AR cAMP responsiveness was assessed by comparing the Δ change in cAMP production between isoproterenol-stimulated and control conditions. Data are shown as median and interquartile range (for β_2_-AR mRNA expression, AY: n = 6; HPA: n = 6, MPA: n = 6 and for cAMP Δ, AY: n = 6; HPA: n = 5, MPA: n = 5). Y = Young, HPA = high physical activity, MPA = moderate physical activity. Kruskal-Wallis test (Group interaction), followed by Mann-Whitney U test were used.

Given the finding that T cells β_2_-AR mRNA abundance does not differ with cell subtype, age, or PA, we next examined isoproterenol-induced β-AR cAMP responsiveness by measuring intracellular cAMP in stimulated T cells. Stimulation with 10 μM isoproterenol produced a significant increase in intracellular cAMP following β-AR stimulation (p = 0.0027, Supplementary Figure S6). There were no significant differences in isoproterenol-induced β-AR cAMP responsiveness (Δ change in cAMP production between isoproterenol-stimulated and unstimulated conditions) across T cells subsets or groups (Figures 4C,D).

## 4 Discussion

It is well established that an acute bout of exercise induces the mobilization and redistribution of lymphocytes, particularly those with a cytotoxic phenotype. This response is β2-AR–dependent and has been linked to enhanced immunosurveillance (Pedersen et al., 2016; Graff et al., 2018; Batatinha et al., 2023; Koivula et al., 2023; Smith et al., 2025). In the present study, we examined the interaction between T cell β2-AR signalling, activation and metabolism among young and older adults who were moderately and highly physically active. We show that individuals with a higher level of habitual PA have lower age-associated impairments in CD8^+^ T cell activation and metabolic switching in response to β stimulation, but this effect was not present with CD4^+^ T cells. While T cell activation and expression of Glut1 increased with PMA/Ionomycin stimulation across groups, β2-AR modulation primarily shaped CD8^+^ T cell activation and glucose transporter, particularly in physically active older adults, which appeared to be largely independent of β2-AR expression or canonical cAMP sensitivity.

The results of the present study show that CD4^+^ T cell activation was maintained with advancing age, whereas CD8^+^ T cell activation was impaired and this was not influenced by physical activity level. Although β-AR stimulation did not enhance CD8^+^ T cell activation following PMA and ionomycin stimulation, the reduction in activation observed with β_2_-AR blockade in the presence of β-AR stimulation suggests that β_1_-AR signaling exerts an inhibitory effect on CD8^+^ T cell activation, independent of age or PA. Together, these findings lead us to hypothesise that during the short-term adrenergic stimulation (30 min in the present study, which represents a bout of exercise), individuals with relatively greater β_1_-AR sensitivity or reduced β_2_-AR responsiveness may exhibit compromised CD8^+^ T cell activation. Interestingly, a study in mice, showed that exhausted CD8^+^ T cells increase ADRB1 expression and exposure of ADRB1^+^ T cells to catecholamines suppressed their cytokine production and proliferation (Globig et al., 2023). The same study also showed that blocking β-adrenergic signalling in CD8^+^ T cells rejuvenated their anti-tumour function, reinforcing the impact of β_1_-AR in CD8^+^ T cell function.

Mechanistically, age-related epigenetic remodeling may contribute to this selective subset-specific impairment. A recent review highlighted that the accumulation of DNA methylation in T cells is a major driver of immune aging and is more pronounced in CD8^+^ than in CD4^+^ T cells (Rousseau et al., 2025), which may partially explain the divergent activation responses observed here. Consistent with this interpretation, Sparks and colleagues demonstrated in a murine model, that aging reduced CD8^+^ T cell number and CD69 expression, whereas CD4^+^ T cells remained largely unaffected (Sparks et al., 2024). Whilst previous studies examining the effects of PA on T cell aging have primarily focused on changes in cell number and phenotypic composition (Duggal et al., 2018; Brauer et al., 2021), most studies have not directly assessed activation outcomes as with the present study. Our findings therefore extend the current literature by identifying a selective age-related vulnerability in CD8^+^ T cell activation that is not influenced by habitual levels of physical activity and may be further modulated by adrenergic receptor signaling.

Stimulation with PMA and ionomycin increased Glut1 expression in both CD4^+^ and CD8^+^ T cells, aligning with extensive literature demonstrating that Glut1 upregulation is a hallmark of T cell activation and is required to support the biosynthetic and energetic demands of effector cell differentiation (Macintyre et al., 2014; Park et al., 2023; Wang et al., 2023). Despite this shared activation response, adrenergic regulation of Glut1 expression was highly subset-specific. In CD4^+^ T cells, Glut1 upregulation was largely independent of β-adrenergic signaling, suggesting that glucose transporter expression in this subset is predominantly driven by activation-dependent signals rather than adrenergic inputs. In contrast, CD8^+^ T cells exhibited a distinct isoproterenol-induced β-AR cAMP responsiveness, whereby β_2_-AR blockade in the presence of β-adrenergic stimulation markedly increased Glut1 expression. Notably, this response was greatest in the HPA group compared to the MPA group and was not different from young participants. Importantly, this observation contrasts with prior work demonstrating that chronic adrenergic stimulation impairs CD8^+^ T cell metabolism and effector function (Slota et al., 2015; Qiao et al., 2019; Farooq et al., 2023). In the present study, however, we examined acute adrenergic exposure (30 min) and show that short-term β-adrenergic signaling when uncoupled from β_2_-AR signaling, enhances metabolic readiness in CD8^+^ T cells, likely through β_1_-AR–dependent mechanisms. This distinction between acute and chronic adrenergic exposure highlights the importance of stimulus duration and signaling context in shaping CD8^+^ T cell metabolic outcomes.

The amplified Glut1 response observed in the HPA group further suggests that adrenergic–metabolic coupling in resting cells (unstimulated) is under control that is lost with β_2_-AR signaling blockade. This improved signaling efficiency may lead to better metabolic flexibility, including a greater increase in glucose transport with activation in CD8^+^ T cells. Consistent with this interpretation, our recent study in the same cohort demonstrated that higher physical activity levels were associated with a reduced hyper-metabolic response common in older adults, with excessive glycolytic dependence and impaired metabolic flexibility (Hazeldine et al., 2025). Supporting this interpretation, the MPA group exhibited enhanced overall Glut1 expression (potentially driven by their higher baseline levels), compared to young controls, whereas this difference was not present in the HPA group. These data suggest that high levels of physical activity are associated with lower glucose transport (as with the young group in this study) which could indicate a higher metabolic reserve, in turns allowing higher metabolic flexibility during activation (van der Windt et al., 2012). However, further functional metabolic measurements would be needed to confirm these hypotheses.

Notably, we did not observe differences in β_2_-AR mRNA abundance or isoproterenol-induced β-AR cAMP responsiveness across the groups, indicating that the observed effects are unlikely to be attributable to changes in receptor abundance or canonical signaling capacity. No effect of age and PA on β_2_-AR mRNA expression indicates that the observed variations in T cells activation and metabolism may result from downstream signaling events, post-transcriptional regulation, or altered receptor sensitivity rather than changes in mRNA abundance. Alternatively, we cannot rule out that more advanced and sensitive techniques might be required to detect subtle differences, since we have not assessed adrenergic receptor expression at the protein level. Indeed, ageing seems to regulate β-adrenergic signaling in other tissues, including heart and blood vessels (Ferrara et al., 2014). This said, the results of related studies seem to be mixed. For example, it has been shown that β_2_-AR binding capacity of lymphocytes from older adults was not significantly different from younger adults (Landmann et al., 1981). However, on the other hand, it has been shown that the immunomodulatory effects of exercise training on lymphocytes from older mice was prevented by β-AR blockade (Kohut et al., 2004). Further, these immunomodulatory effects were less affected by β_2_-AR blockade in younger mice, suggesting older mice were more dependent of their β-AR signaling to sustain a robust immune response. Together, these findings suggest that aging may increase reliance on β-adrenergic signaling to support immune cell activation and metabolic responsiveness. In the context of our study, this framework may help explain why β-AR modulation had a more pronounced influence on CD8^+^ T cell activation and Glut1 expression despite no detectable differences in receptor expression or basal signaling sensitivity. Rather than reflecting changes in receptor abundance, our results support the interpretation that β_2_-AR signaling is associated with age- and physical activity–dependent regulation of CD8^+^ T cell activation and metabolic phenotype.

No differences were observed in lipid accumulation and there was no increase within groups during stimulation. A previous study has also shown no change in lipid accumulation assessed by BODIPY™ in CD4^+^ T cells (Noureddine et al., 2024), after 6 h of anti-CD2/anti-CD3/anti-CD28 stimulation. In the present study, almost no change in glucose utilization was observed, except for a reduction in 2-NBDG in CD4^+^ T cells from young participants, but no difference between groups. Although the use of 2-NBDG as a surrogate marker of glucose uptake has been widely accepted, the use of 2-NBDG uptake alone has considerable limitations, at least in T cells (Sinclair et al., 2020) and therefore this result should be interpreted with caution. Therefore, we additionally examined Glut1 expression as a marker of metabolic activity. Although Glut1 expression is commonly used as a marker of activation-induced metabolic reprogramming via increased glucose uptake, elevated Glut1 levels do not necessarily indicate increased metabolic activity, as glucose utilization also depends on transporter localization, co-stimulatory signaling, nutrient availability, and downstream metabolic pathways (Michalek et al., 2011). The combination of PMA and Ionomycin stimulation leads to PKC activation and Ca^2+^ influx, engaging metabolic regulators such as Myc, Akt and mTOR pathways. Although this led to a robust increase in Glut1 expression, it is possible that 4 h of stimulation were not enough to promote significant changes in glucose utilization and lipid accumulation (Macintyre et al., 2014).

### 4.1 Limitations

It should be acknowledged that in the present study, 30 min of isoproterenol as a β-adrenergic ligand to mimic an acute bout of exercise. However, during exercise, β-adrenergic receptors will be stimulated by adrenaline and noradrenaline that will also bind to α-receptors (Williams et al., 1976). It is noteworthy that α-adrenergic receptors were not considered in this study, given their very low density on T cells (Williams et al., 1976). The β_2_ receptor blockade with ICI-118,551 would also benefit from further validation comparing different concentrations to support its selectivity in this type of cell (Baker, 2005). Future studies should distinguish between the effects of β_1_ and β_2_-AR receptors, or the influence of other receptors in the regulation of T cell activation and metabolism. Furthermore, aside from β-adrenergic stimulation, there are other neurotransmitters, hormones, inflammatory factors and hemodynamic changes that occur simultaneously during exercise that were not considered in the present study and could be incorporated into the design of future experiments. It is also worth considering that stimulation with anti-CD3/anti-CD28 would be a more physiological form of stimulation (that partly mimics antigenic stimulation) compared to PMA and ionomycin. This, the conclusions of this study are limited to pharmacologically induced activation. However, anti-CD3/anti-CD28 would require longer-term stimulation (at least 48 h) which in turn would lead to desensitization or adaptation of β_2_-adrenergic signaling *in vitro*. Such a lengthy incubation would complicate interpretation of the short-term effects of isoproterenol from downstream chronic responses.

Another limitation of the present study was that we did not include a true positive control for the glucose utilization and lipid accumulation assays. Although PMA and ionomycin stimulation are used as positive control in other studies to stimulate cytokine production in T cells, a longer duration would be necessary to generate significant changes in glucose utilization and lipid accumulation, specifically. Also, the lack of vehicle-control comparison also add the possibility of the effects observed to be cause by the vehicles used, such as HBSS and DMSO; however to prevent its negative effects we worked with serial dilutions and final RPMI dilutions that results in less than 2% of these vehicles in the experiments. Together with the limited metabolic and functional readouts of this study, the results should be considered hypothesis-generating rather than definitive.

More broadly, a significant limitation of the present study is the small sample size, therefore our findings require replication in a larger study. In addition, although the comparison of habitual PA between older adults was rigorously analysed by 7-days of accelerometery, comparisons between young and older adults were undertaken using data from a questionnaire that was not validated. Furthermore, all older adults were physically active, accumulating more than typical physical activity recommendations, which limited our ability to examine physically active compared to physically inactive individuals. Nonetheless, we were able to stratify older adults into two groups, in which one of them (MPA) achieved roughly half of the volume of physical activity compared to the other (HPA). It is also worth considering that although there is limited evidence for sex differences in the density or sensitivity of β_2_AR in T cells, estrogen can influence cAMP signaling, cytokine production, and differentiation in T cells, including their sympathetic responsiveness (Fan and Wang, 2009), it is possible that sex-specific responses to the same catecholamine concentration, could be a potential confounding factor in the results of the present study. However, only one male participant in one group (MPA) compared to two males in the other group (HPA), limited further analysis. In addition, aging and physical activity also modify the numbers and proportions of T cell subsets (naïve, memory, senescent) (Duggal et al., 2019), and therefore different metabolic and adrenergic readouts from each subset could be masked when considering the overall CD4^+^ and CD8^+^ T cell populations.

## 5 Conclusion

Due to many limitations, we emphasise that this is a hypothesis-generating study and future studies will be needed to generate definitive results. Overall, these findings provide new insight into how aging, physical activity, and adrenergic signaling interact to shape T cell pharmacological activation and metabolic responses under conditions relevant to acute physiological stress. While CD4^+^ T cell activation and metabolic upregulation appeared largely resilient to adrenergic modulation, CD8^+^ T cells showed greater isoproterenol-induced β-AR cAMP responsiveness, with physical activity being associated with how these cells integrate metabolic cues without restoring age-related impairments in activation. Several studies have reported that habitual physical activity improves T cell function, reduces the risk of chronic diseases and acute respiratory infections (Duggal et al., 2019; Grande et al., 2020). However, how long-term physical activity shapes β_2_-AR signaling and T cell metabolism has remained unclear. The present work helps address this gap by demonstrating that adrenergic-metabolic coupling in T cells differs in older adults with different PA levels, highlighting metabolic integration, as opposed to receptor expression or isoproterenol-induced β-AR cAMP responsiveness, as a key determinant of immune responsiveness with aging. Furthermore, since some responses modified by β stimulation do not depend on β_2_-AR, one hypothesis that deserves future investigation is the potential participation of β_1_-AR in this modulation to generate definitive conclusion.

## Data Availability

The raw data supporting the conclusions of this article will be made available by the authors, without undue reservation.
