# Supplementary material for: Impact of habitual physical activity and age on T cell metabolism and activation via β2-adrenergic receptor modulation
Source: Front Aging. 2026 Oct 20;7:1869552. doi: 10.3389/fragi.2026.1869552 (PMC13727592; doi:10.3389/fragi.2026.1869552)
Supplement: Supplementary file 1 [file Supplementaryfile1.docx]

**Impact of Habitual Physical Activity and Age on T cell Metabolism and Activation via β_2_-Adrenergic Receptor Modulation**

**Supplementary material**

***Supplementary Figure 1.*** *Daily MVPA in MPA and HPA groups. MVPA was measured using 7-day accelerometry. Data is presented as median and range (MPA: n = 6, HPA: n = 6). Statistical analysis was performed using an independent samples t-test. p = 0.0004. MPA = moderate physical activity, HPA = high physical activity.*

***Supplementary Figure 2.*** *Schematic representation of the experimental design that allowed calculation of delta MFI values for CD69 and GLUT1.*

***Supplementary Figure 3.*** *Histogram for CD69 MFI under unstimulated (blue) and stimulated conditions with PMA and Ionomycin (red). Left panel: CD4+ T cells. Right panel: CD8+ T cells.*

***Supplementary Figure 4.*** *Histogram for GLUT1 MFI under unstimulated (blue) and stimulated conditions with PMA and Ionomycin (red). Left panel: CD4+ T cells. Right panel: CD8+ T cells.*

**Supplementary Table 1.** *CD69 and GLUT1 positive cells within CD4+ and CD8+ T cells (from PBMCs) under the six treatment conditions.*

| **CD69+ (%)** | | **MPA** | | **HPA** | | | | **Young** | | | | **P- values** | | | | | |  |
| --- | --- | --- | --- | --- | --- | --- | --- | --- | --- | --- | --- | --- | --- | --- | --- | --- | --- | --- |
|  | | **Control** | **Stimulation** | **Control** | | **Stimulation** | | **Control** | | **Stimulation** | | **Interaction** | | **Group** | | **Condition** | |  |
| CD4+CD69+ | | 6.55 ± 2.27 | 54.62 ± 9.16 | 6.67 ± 2.46 | | 57.68 ± 12.49 | | 9.13 ± 4.73 | | 64.47 ± 10.77 | | 0.579 | | 0.209 | | **0.000** | |  |
| CD8+CD69+ | | 11.15 ± 2.61 | 48.72 ± 12.11 | 13.42 ± 9.07 | | 47.4 ± 10.38 | | 16.56 ± 7.16 | | 67.35 ± 12.32 | | **0.032a** | | **0.049** | | **0.000** | |  |
| **Control** | | **ISO** | **BLOCK** | **ISO** | | **BLOCK** | | **ISO** | | **BLOCK** | | **Interaction** | | **Group** | | **Condition** | |  |
| CD4+CD69+ | | -0.03 ± 0.45 | 6.02 ± 3.34 | 0.46 ± 1.4 | | 15.66 ± 31.36 | | -0.02 ± 0.75 | | 4.97 ± 3.25 | | 0.603 | | 0.544 | | 0.070 | |  |
| CD8+ CD69+ | | 0.43 ± 0.37 | 2.18 ± 1.89 | 0.84 ± 2.02 | | 4.56 ± 13.77 | | -0.16 ± 0.81 | | 0.97 ± 3.31 | | 0.856 | | 0.666 | | 0.284 | |  |
| **Stimulated** | | **ISO** | **BLOCK** | **ISO** | | **BLOCK** | | **ISO** | | **BLOCK** | | **Interaction** | | **Group** | | **Condition** | |  |
| CD4+CD69+ | | -0.06 ± 3.46 | 5.86 ± 4.86 | -4.78 ± 16.59 | | 5.26 ± 8.91 | | 5.47 ± 8.73 | | 15.57 ± 13.57 | | 0.853 | | 0.122 | | **0.024** | |  |
| CD8+CD69+ | | 0.2 ± 1.99 | -7.48 ± 7.26 | -5.84 ± 14.7 | | -9.84 ± 10.04 | | 2.45 ± 7.76 | | -1.22 ± 11.47 | | 0.899 | | 0.093 | | 0.212 | |  |
|  | | | |  | |  | |  | |  | |  | |  | |  | |  |
| **GLUT1+ (%)** | | **MPA** | | **HPA** | | | | **Young** | | | | **P- values** | | | | | |  |
|  | | **Control** | **Stimulation** | **Control** | | **Stimulation** | | **Control** | | **Stimulation** | | **Interaction** | | **Group** | | **Condition** | |  |
| CD4+GLUT1+ | | 6.98 ± 4.18 | 29.49 ± 31.6 | 4.52 ± 0.8 | | 31.12 ± 29.08 | | 3.97 ± 2.78 | | 11.76 ± 5.98 | | 0.365 | | 0.348 | | 0.005 | |  |
| CD8+GLUT1+ | | 53.22 ± 11.91 | 76.74 ± 18.95 | 48.62 ± 6.71 | | 77.16 ± 7.32 | | 31.37 ± 11.2 | | 64.22 ± 17.61 | | 0.644 | | **0.033b** | | 0.000 | |  |
| **Control** | | **ISO** | **BLOCK** | **ISO** | | **BLOCK** | | **ISO** | | **BLOCK** | | **Interaction** | | **Group** | | **Condition** | |  |
| CD4+GLUT1+ | | 0.63 ± 1.35 | 2.8 ± 2.15 | 10.63 ± 25.48 | | 2.31 ± 1.38 | | -0.37 ± 2 | | 1.33 ± 3.25 | | 0.399 | | 0.429 | | 0.669 | |  |
| CD8+GLUT1+ | | -3.04 ± 7.39 | 6.8 ± 9.02 | -1.42 ± 3.98 | | 15.16 ± 5.77 | | -1.53 ± 6.14 | | 8.52 ± 5.22 | | 0.153 | | 0.391 | | **0.000** | |  |
| **Stimulated** | | **ISO** | **BLOCK** | **ISO** | | **BLOCK** | | **ISO** | | **BLOCK** | | **Interaction** | | **Group** | | **Condition** | |  |
| CD4+GLUT1+ | | 12.63 ± 17.73 | 4.94 ± 5.15 | -9.08 ± 16.04 | | -7.3 ± 14.06 | | 7.27 ± 7.12 | | -2.86 ± 2.41 | | 0.187 | | 0.062 | | 0.062 | |  |
| CD8+GLUT1+ | | 1.28 ± 3.05 | 5.58 ± 2.51 | -0.96 ± 5.88 | | 6.64 ± 10.57 | | -5.58 ± 9.15 | | -2.03 ± 18.69 | | 0.861 | | 0.297 | | 0.130 | |  |
|  | |  |  |  | |  | |  | |  | |  | |  | |  | |  |

***Legend:*** *Data is presented as mean ± standard deviation. ISO= β adrenergic stimulation with Isoproterenol (1 μM); BLOCK****=*** *β adrenergic stimulation with Isoproterenol (1 μM) and β₂ blockade with ICI-118,551 (300 nM); a= MPA and HPA significant different from young with stimulation (PMA and Ionomycin); b= MPA different from Young.*

**Supplementary Figure 5.** Gating strategy for the percentage of CD69+ cells and GLUT1+ cells among CD4+ and CD8+ T cells.

**Supplementary Figure 6.** Gating strategy showing median fluorescence intensity (MFI) for 2-NBDG among CD4+ and CD8+ T cells.

***Supplementary Figure 7.*** *Gating strategy for median fluorescence intensity (MFI) of Bodipy^TM^ among CD4+ and CD8+ T cells.*

***Supplementary Figure 8.*** *Luminescence levels in isolated CD4⁺ and CD8⁺ T cells that are unstimulated and stimulation with 10 µM isoproterenol, expressed as RLU/µg/ml (inversely related to cAMP concentration). Data are presented as Whiskers: Min-Max (n =16). Statistical analysis was performed using a paired samples t-test. p = 0.0027.*

***Supplementary Figure 9.*** *GLUT1 expression in CD69+* T cells *(from PBMCs) in 6 treatment conditions*. *Figure 1 A-B: GLUT1 MFI on CD4+ and CD8+ T cells with and without PMA/ionomycin stimulation. Figure 1 C-D: Δ GLUT1 MFI on CD4+ and CD8+ T cells calculated as the difference between GLUT1 expression in control tubes (no PMA/ionomycin stimulation): isoproterenol – untreated control and ICI-118,551 + isoproterenol – untreated control. Figure 1 E-F: Δ GLUT1 MFI on CD4+ and CD8+ T cells calculated as the difference between GLUT1 expression in stimulated tubes (PMA/ionomycin stimulation): (isoproterenol + PMA/ionomycin) – PMA/ionomycin alone and (ICI-118,551 + isoproterenol + PMA/ionomycin) – PMA/ionomycin alone. Data are shown as median fluorescence intensity (MFI) and interquartile range of MFI across all donors (Y: n = 6; HPA: n = 5, MPA: n = 5). Y = Young, HPA = high physical activity, MPA = moderate physical activity.*
